# New Developments in Mammalian Target of Rapamycin Inhibitors for the Treatment of Sarcoma

**DOI:** 10.1002/cncr.26361

**Published:** 2011-08-11

**Authors:** Mark Agulnik

**Affiliations:** Division of Medical Hematology/Oncology, Northwestern UniversityChicago, Illinois

**Keywords:** sarcoma, mammalian target of rapamycin (mTOR) protein, sirolimus, temsirolimus, ridaforolimus, everolimus

## Abstract

Although sarcomas account for a small portion of solid malignancies, currently, there are few treatment options for sarcomas, particularly for advanced disease. The mammalian target of rapamycin (mTOR), a serine-threonine protein kinase in the phosphatidylinositol 3-kinase/serine/threonine protein kinase Akt signaling pathway, has an important role in the regulation of protein synthesis, cell proliferation, angiogenesis, and metabolism. Alterations of the mTOR signaling pathway are common in malignancies, including several types of sarcoma. Therefore, mTOR is a potentially important therapeutic target in these diseases. Rapamycin and its analogs (rapalogs) are effective anticancer agents in a broad range of preclinical models. Clinical trials with these agents alone and in combination with other anticancer agents, including chemotherapy and targeted therapies, have demonstrated potential clinical benefit in several types of sarcoma. The evidence from both preclinical and clinical studies supports further study of mTOR-targeting rapalogs in the treatment of various subtypes of sarcoma. Cancer 2011;. © 2011 American Cancer Society.

## INTRODUCTION

Sarcomas are a group of heterogeneous tumors that originate from mesenchymal tissue. In the United States, sarcomas account for approximately 1% of all adult solid malignancies and approximately 15% of pediatric cancers.^1^ In 2010, an estimated 13,170 new cases of soft tissue and bone sarcomas were diagnosed, resulting in 5380 deaths in the United States.^2^, ^3^ Currently, few options exist for the treatment of sarcomas. Standard therapy includes surgery, chemotherapy, and radiotherapy; the most frequently used treatment for advanced disease is chemotherapy with anthracyclines (eg, doxorubicin), alkylating agents (eg, ifosfamide and dacarbazine), and platinum compounds (eg, cisplatin and carboplatin), or combinations of these agents.^4^ Gastrointestinal stromal tumors (GISTs) are a unique subtype of soft tissue sarcoma (STS) and can be treated with surgery and/or tyrosine kinase inhibitors, including imatinib and sunitinib; the primary therapy for advanced metastatic/unresectable GIST is imatinib.^1^

Aberrant activity in several molecular pathways has been linked to the pathogenesis of various sarcoma subtypes. Mahalingam et al recently published an in-depth review of the molecular alterations in sarcomas, which include the up-regulation or mutational activation of receptor tyrosine kinases (KIT, insulin-like growth factor 1 receptor [IGF-1R], epidermal growth factor receptor [EGFR], and platelet-derived growth factor receptor) and members of the phosphatidylinositol 3-kinase (PI3K)/threonine protein kinase Akt (Akt)/mammalian target of rapamycin (mTOR) pathway; loss or deletions of tumor suppressor genes (eg, retinoblastoma, p53, and phosphatase and tensin homolog [PTEN]); increased vascular endothelial growth factor (VEGF) pathway expression and angiogenesis; mutations, amplification, or overexpression of oncogenes (eg, the v-myc myelocytomatosis viral oncogene homolog [c-Myc], Ras, and the v-src sarcoma viral oncogene homolog [Src]); and dysregulation of apoptosis through B-cell chronic lymphocytic leukemia/lymphoma 2 (Bcl-2) overexpression.^4^ The characterization of these molecular pathways has led to the development of novel targeted biologic therapies as treatment options.

Mammalian target of rapamycin, a serine/threonine kinase that has a pivotal role in the control of cell growth, metabolism, cell proliferation, and cell survival through the PI3K/Akt/mTOR pathway, is considered an important target for anticancer drug development.^5^-^8^ Although sirolimus (rapamycin), the first mTOR inhibitor discovered, initially was developed as an immunosuppressive agent, preclinical studies in both in vitro and xenograft models have demonstrated that sirolimus inhibits the growth of several murine and human cancer cell lines.^9^ On the basis of these results, further studies have examined the potential role of sirolimus as an anticancer agent. Derivatives of rapamycin with improved pharmacokinetics and reduced immunosuppressive effects have been developed (ie, temsirolimus, everolimus, and ridaforolimus) and currently are under clinical investigation.^9^, ^10^ This article briefly describes the mTOR pathway and its role in cancer and reviews data from preclinical and clinical studies of mTOR inhibitors, specifically those being investigated in sarcoma.

### The Mammalian Target of Rapamycin Pathway

**Figure 1:**
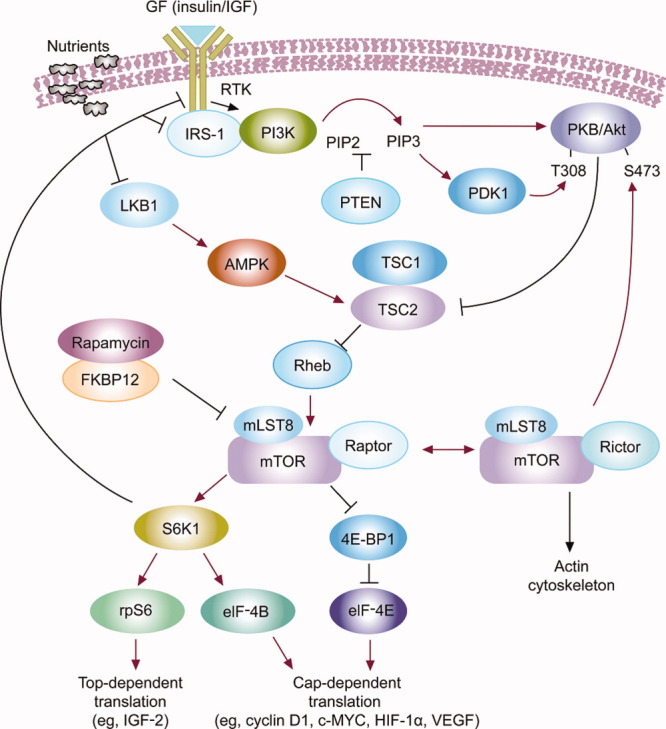
The mammalian target of rapamycin (mTOR) signaling cascade and its function are illustrated. GF indicates growth factor; IGF, insulin-like growth factor; RTK, receptor tyrosine kinase; IRS-1, insulin receptor substrate 1; PI3K, phosphatidylinositol 3 kinase; PIP, phosphatidylinositol-4,5-bisphosphate; PKB, phosphate kinase B; LKB1, serine threonine kinase 11; PTEN, phosphatase and tensin homolog (deleted on chromosome 10); PDK1, 3-phosphoinositide-dependent protein kinase 1; T308, threonine 308; S473, serine 472; AMPK, adenine monophosphate-activated kinase; TSC, tuberous sclerosis complex; Rheb, Ras homolog enriched in brain; FKBP12, 12-kDa FK506-binding protein; mLST8, G protein subunit like (mTOR complex subunit LST8); S6K1, protein S6 kinase 1; 4E-BP1, eukaryotic initiation factor 4E binding protein-1; rpS6, ribosomal protein S6; elF-4E, eukaryotic initiation factor 4E; HIF-1α, hypoxia inducible factor 1α; VEGF, vascular endothelial growth factor.

Mammalian target of rapamycin is a member of the PI3K-kinase related kinase superfamily.^11^, ^12^ Human mTOR exists in 2 different multiprotein complexes: mTOR complex 1 (mTORC1), consisting of mTOR, mTOR complex subunit LST8 (mLST8), and regulatory-associated protein of mTOR (raptor); and mTOR complex 2 (mTORC2), composed of mTOR, mLST8, rapamycin-insensitive companion of mTOR (rictor), and mammalian stress-activated protein kinase-interacting protein 1 (mSin1).^12^ Of the 2 complexes, mTORC1 has been studied more extensively and reportedly regulates most mTOR effects on protein synthesis and gene expression associated with cell growth, metabolism, cell proliferation, angiogenesis, and cell survival. The role of mTORC2 is less understood, but reports suggest that mTORC2 phosphorylates Akt in the PI3K/Akt pathway and regulates the organization of the cytoskeleton (Fig. .1).^6^-^8^, ^13^, ^14^ The activity of mTOR is regulated by growth factors and their receptors, which transmit signals through the PI3K/Akt and Ras pathways.^9^ Members of the EGFR family (eg, EGFR, human epidermal growth factor 2), IGF, and VEGF receptors stimulate mTOR activity through the small guanosine triphosphatase (GTPase) Ras homolog enriched in brain.^9^ Signals generated by these receptors are regulated by PTEN, which inhibits PI3K signaling; neurofibromatosis type-1 (NF1), a tumor suppressor that reduces Ras activity; and tuberous sclerosis complex (TSC1) and TSC2, which form a complex to block the activation of mTOR.^11^ The activity of mTOR also is regulated by cellular stress—when intracellular adenosine triphosphate (ATP) levels are depleted, the adenosine monophosphate-activated protein kinase is activated through the tumor suppressor LKB1 (serine threonine kinase 11). Adenosine monophosphate-activated protein kinase subsequently activates another tumor suppressor, TSC1/TSC2, thereby leading to mTOR inactivation.^10^, ^14^

The activation of mTOR stimulates at least 2 downstream effectors: 4E-binding protein 1 (4E-BP-1)/4E-BP-2 and ribosomal protein S6 kinases 1 and 2, which function in translational control to regulate mammalian cell size.^15^ Mammalian target of rapamycin signaling leads to the expression of several proteins^12^, ^16^-^18^: c-MYC, cyclin D, and ornithine decarboxylase, which is involved in the G1 to S transition during cell proliferation^7^; hypoxia-inducible factor-1α (HIF-1α), which is involved with metabolism and angiogenesis^19^, ^20^; VEGF and fibroblast growth factor (FGF), which also are associated with angiogenesis^19^, ^20^; ribosomal proteins, poly(A)-binding protein, and elongation factors, which are part of the cellular translational machinery involved in protein synthesis and ultimately cell growth^17^, ^18^, ^21^; and the growth factor IGF-2.^12^, ^22^ Because of the complex network of downstream effects linked to the activation of mTOR, dysregulation of the pathway is linked to several malignancies.^9^

### Mammalian Target of Rapamycin Up-Regulation and Down-Regulation in Sarcoma

Abnormal mTOR activity, including the dysregulation of members of its pathway (such as growth factor receptors and tumor suppressors),^9^ has been documented in several tumor types, including colorectal, lung, and breast cancers.^21^, ^23^ Overexpression of growth factor receptors or mutation of their associated receptor tyrosine kinases leads to increased signaling through the PI3K/Akt/mTOR pathway. In some sarcoma subtypes, specifically rhabdomyosarcoma, leiomyosarcoma, Ewing sarcoma, synovial sarcoma, and osteosarcoma, members of the epidermal growth factor family (including IGF-1R and its ligands, IGF-1 and IGF-2) reportedly are overexpressed.^12^, ^24^-^26^ The up-regulation of other receptor tyrosine kinases, such as FGF receptor and EGFR, also have been reported in Ewing sarcoma, rhabdomyosarcoma, and osteosarcoma.^12^, ^26^-^28^ Deletions of the tumor suppressors TSC1/TSC2 and NF1 are associated with both benign sarcoma-like tumors, such as angiomyolipomas, lymphangioleiomyomatosis, rhabdomyomas, neurofibromas, hamartomas, and schwannomas, and malignant sarcomas, such as malignant peripheral nerve sheath tumors.^14^, ^29^-^32^ Because of the key role of mTOR in regulating these pathways, the inhibition of mTOR has become a desirable therapeutic option in the treatment of cancer.

### Mammalian Target of Rapamycin Inhibitors Under Investigation in Cancer Treatment

Several mTOR inhibitors currently are under investigation for possible therapeutic use in the treatment of cancer, including sarcomas. These include sirolimus and its analogs temsirolimus, everolimus, and ridaforolimus. Sirolimus, also known as rapamycin, is the prototype mTOR inhibitor—it is a natural compound that initially was approved as an immunosuppressant for organ transplantation but also is known for its antifungal and anticancer activities.^10^, ^33^, ^34^ In cancer, sirolimus may alter the composition and/or conformation of the multiprotein mTOR complexes and allosterically block access of substrates to the mTOR kinase domain by binding to the kinase domain of mTOR.^10^ This causes an inhibition of cell proliferation by arresting cells in the G1 phase and inducing apoptosis in selected models.^10^ However, initially, it was speculated that the ability to use sirolimus at effective doses as an anticancer agent would be hindered by reports of poor aqueous solubility and chemical stability.^5^ In an effort to improve on the natural sirolimus product, novel analogs have been created.^34^ Temsirolimus, a prodrug of sirolimus, is a selective binding inhibitor of mTOR that acts on a variety of tumor cells, in particular those with a PTEN deletion.^35^, ^36^ Everolimus, an orally available mTOR inhibitor with greater solubility than sirolimus, was developed in an attempt to improve the pharmacokinetic characteristics of sirolimus, particularly to increase oral bioavailability.^37^ Ridaforolimus is a nonprodrug analog of sirolimus with favorable pharmacokinetic properties, including solubility, stability, and bioavailability.

Only temsirolimus (Torisel; Pfizer, New York, NY) and everolimus (Afinitor; Novartis, East Hanover, NJ) have Food and Drug Administration-approved indications in oncology for the treatment of advanced renal cell carcinoma.^38^, ^39^ Although sirolimus is currently not indicated for the treatment of cancer, the National Comprehensive Cancer Network guidelines recommend its use for the treatment of angiomyolipoma and lymphangioleiomyomatosis as well as perivascular epithelioid cell tumors.^1^ Ridaforolimus, temsirolimus, and everolimus are being investigated for use in sarcoma treatment.^22^, ^40^ There are ongoing phase 2 trials for sirolimus, temsirolimus, everolimus, and ridaforolimus; also, a phase 3 trial for ridaforolimus as maintenance therapy in sarcoma has completed enrollment.

### Preclinical Studies

#### Sirolimus

Sirolimus has demonstrated ability to inhibit the growth of B16 melanoma, P388 leukemia, MiaPaCa-2 cells, and Panc-1 human pancreatic carcinoma in xenograft models.^41^, ^42^ Results from the Pediatric Preclinical Testing Program have indicated that sirolimus has broad antitumor activity against in vivo panels of childhood tumors, with noteworthy activity in select sarcoma and acute lymphoblastic leukemia xenografts.^43^ In addition, recent data from this program, which examined in vivo solid tumor models, including sarcomas (Ewing, osteosarcoma, rhabdomyosarcoma), have demonstrated the therapeutic potential of sirolimus in combination with cytotoxic agents such as cyclophosphamide or vincristine.^44^

#### Temsirolimus (CCI-779)

In murine xenograft models of rhabdomyosarcoma cell lines (Rh30 and RD), treatment with temsirolimus was effective in inhibiting tumor growth.^45^ The antitumor activity of temsirolimus was associated with a reduction of HIF-1α levels and VEGF protein expression and with decreased microvessel density in Rh30-derived and RD-derived tumors, demonstrating suppressed tumor growth through an antiangiogenic mechanism. Treatment with a single 20-mg/kg dose of temsirolimus suppressed the phosphorylation of S6 and 4E-BP1, indicating the inhibition of mTOR activity. In another study that used a rhabdomyosarcoma Rh30 mouse xenograft model, a high correlation coefficient was reported between decreases in phosphorylation of threonine residue 70 (Thr^70^) of 4E-BP1 and tumor growth inhibition with temsirolimus.^35^ These results suggest that decreases in Thr^70^ phosphorylation of 4E-BP1 may be a useful surrogate marker for determining inhibition of mTOR activity in tumors.

#### Everolimus (RAD001)

Everolimus has demonstrated antiproliferative activity against several tumor cell lines and in a broad range of human tumor xenografts.^46^-^53^ In a mouse model of human GIST, everolimus inhibited translational response and cell proliferation in tumor lesions.^54^ By virtue of its ability to induce cell cycle arrest, these results suggest that everolimus is potentially useful in the treatment of patients with imatinib-resistant GIST. Treatment with everolimus also decelerated tumor growth and prolonged life span in a mouse model of leiomyosarcoma.^55^

#### Ridaforolimus (AP23573, MK-8669)

Studies of ridaforolimus in human xenograft models of various tumor cell lines (prostate, breast, pancreas, lung, and colon) have demonstrated potent inhibition of tumor growth.^56^ Ridaforolimus also reduced the rate of cell proliferation in vitro in a panel of 11 sarcoma and 6 endometrial cell lines and inhibited the rate of tumor growth in a leiomyosarcoma xenograft model.^57^ In another study, sarcoma and endometrial cancer cell lines were treated in vitro with combinations of drugs that included ridaforolimus to determine their antagonistic, additive, or synergistic effects on cell growth.^58^ The combination of ridaforolimus and doxorubicin demonstrated at least an additive inhibitory effect in 4 sarcoma cell lines. The combination of ridaforolimus with doxorubicin, carboplatin, or paclitaxel as well as the triple combination of carboplatin, paclitaxel, and ridaforolimus had additive effects on 3 endometrial cell lines. Additive growth inhibition of all sarcoma and endometrial cancer cell lines also was observed when ridaforolimus was combined with 2-deoxyglucose, a metabolic inhibitor.

### Phase 1 Studies

The design of phase 1 studies of mTOR inhibitors generally has followed that of phase 1 studies for cytotoxic agents, including the determination of safety and tolerability and definition of the maximum tolerated dose (MTD).

#### Sirolimus

In patients (N = 21) with solid tumors (eg, sarcoma, pancreatic, colorectal, hepatocellular, and neuroendocrine tumors), the MTD of oral sirolimus was 6 mg daily.^59^ No objective responses were observed, but 10 patients achieved stable disease (SD). Results from that study indicated that drug exposure increased in proportion to dose and that the pharmacokinetic profile of sirolimus was comparable to that in transplantation studies. These results suggest that, in contrast to previous reports, sirolimus is sufficiently absorbed and, thus, may be an effective mTOR inhibitor for cancer therapy.^59^ Currently, phase 1 studies are evaluating oral sirolimus for the treatment of patients with human immunodeficiency virus-related Kaposi sarcoma^60^ and in combination with bevacizumab for the treatment of advanced solid tumors.^61^ Finally, a phase 1 study is assessing nanoparticle albumin-bound-rapamycin (ABI-009) in patients with advanced solid tumors, including sarcoma.^62^, ^63^

#### Temsirolimus

In a phase 1 trial, intravenous temsirolimus was administered to patients (N = 63) with advanced cancer (eg, solid tumors, including sarcomas or lymphomas). The MTD was 15 mg/m^2^ daily for 5 days every 2 weeks for patients who had received extensive previous anticancer treatment and 19 mg/m^2^ daily for 5 days every 2 weeks for minimally pretreated patients.^64^ In another study, patients (N = 24) who received a weekly intravenous temsirolimus dose (7.5-220 mg/m^2^) demonstrated antitumor activity; confirmed partial responses (PRs) were evident in 2 patients, and minor responses were reported in 2 additional patients.^65^ Furthermore, another phase 1 study examining an oral formulation of temsirolimus in patients with advanced cancer (N = 24) reported an MTD of 75 mg once daily for 5 days every 2 weeks. The most common treatment-related adverse events (AEs) were mucositis, rash/maculopapular rash, and asthenia.^66^

Other ongoing phase 1 trials are evaluating the combination of intravenous temsirolimus with sorafenib, a tyrosine kinase inhibitor, in patients with advanced solid tumors^67^; valproic acid in young patients with relapsed neuroblastoma, bone sarcoma, or STS^68^; vinorelbine for advanced solid tumors, including uterine sarcoma^69^; liposomal doxorubicin in patients with recurrent sarcoma^70^; and irinotecan for patients with refractory sarcomas.^71^

#### Everolimus

Two studies were conducted in patients with advanced tumors (eg, colorectal, nonsmall cell lung, pancreas, and breast) who were unresponsive to standard therapy. In 1 trial, oral everolimus was tolerated by patients (N = 55) at a dose of 10 mg daily or 50 mg per week; whereas, in the other trial, oral everolimus was tolerated by patients (N = 92) at doses up to 10 mg daily and 70 mg per week.^72^, ^73^ The results demonstrated that everolimus is dose dependent and that continuous daily dosing produced more profound mTOR inhibition than weekly dosing. Those studies also demonstrated a clinical benefit with oral everolimus: In 1 study, a clinical benefit was observed in 4 patients; and, in the second study, PRs were observed in 4 patients, and 12 patients remained progression-free for ≥6 months.^72^, ^73^ Moreover, an ongoing study is evaluating oral everolimus in combination with vatalanib, a tyrosine kinase inhibitor, in patients with advanced solid tumors.^74^ In pediatric patients, a phase 1 trial has investigated oral everolimus for the treatment of recurrent or refractory solid tumors, including STS,^75^ whereas other trials (phase 2) are planned to further evaluate treatment of advanced sarcoma.^76^, ^77^

#### Ridaforolimus

Two phase 1 studies examined patients with solid malignancies (eg, STS, renal cell carcinoma, nonsmall cell lung carcinoma, and transitional cell carcinoma of the bladder) using the intravenous formulation of ridaforolimus.^78^, ^79^ In 1 trial, in patients (N = 32) who received at least 1 dose of ridaforolimus (3-28 mg daily), the MTD for ridaforolimus was 18.75 mg intravenously once daily for 5 consecutive days every 2 weeks.^78^ Among the patients who were evaluated for tumor response, 22 achieved SD or a PR; all patients with sarcoma and renal cell carcinoma experienced a PR, SD, or a minor response that lasted for >3 months. In the other trial, in patients (N = 46) who received various doses of ridaforolimus (6.25-225 mg daily), the MTD was 75 mg per week.^79^ Of 30 patients who were evaluable for response to daily ridaforolimus treatment, 22 achieved SD, and 7 had a best overall response to disease progression. On the basis of these results, the dose recommended for phase 2 trials was 12.5 mg intravenously once daily for 5 days every other week.^11^

Oral regimens of ridaforolimus also have been examined in patients (N = 147) with advanced metastatic solid tumors refractory to standard therapy.^80^ A ridaforolimus dose of 40 mg once daily for 5 days each week demonstrated antitumor activity consistent with the intravenous formulation.^80^ Clinical benefit (defined as SD for >16 weeks, a PR, or complete response [CR]) was observed with all regimens in patients with several types of sarcomas and a variety of carcinomas; 36 patients (24.5%) achieved a clinical benefit, including 23 patients with sarcoma (15.6%). In the group that received ridaforolimus 40 mg once daily for 5 days each week (n = 24), 3 of 13 patients (23%) with sarcomas achieved a clinical benefit, and 2 of 13 patients (15.4%) achieved a PR. The 6-month progression-free survival (PFS) rate was 29%, and the median PFS was 17 weeks for patients with sarcoma with all regimens.^80^ The intravenous formulation of ridaforolimus also has been investigated in pediatric patients with advanced solid tumors.^81^

Other phase 1 trials have examined intravenous ridaforolimus in combination with paclitaxel (a cytotoxic agent) for the treatment of taxane-sensitive solid tumors^82^ and oral ridaforolimus in combination with bevacizumab (a monoclonal antibody that blocks VEGF-A) for patients with advanced cancers.^83^ Currently, oral ridaforolimus is being studied in combination with standard chemotherapy for STS.^84^

### Phase 2 Studies

#### Sirolimus

A phase 2, nonrandomized, open-label trial investigated the treatment of angiomyolipoma, a benign renal neoplasm rich in fat, muscle, and blood vessels, with sirolimus in patients (N = 25) with TSC or sporadic lymphangioleiomyomatosis (Table 1).^86^ The results indicated that oral sirolimus reduced the volume of renal angiomyolipomas, and tumors regressed during therapy but generally increased in volume after cessation of treatment. The majority of patients experienced an AE, and 5 patients experienced serious AEs (SAEs) while taking sirolimus. In another phase 2 trial, oral sirolimus was evaluated in patients with complicated vascular anomalies, including Kaposi-form hemangioendotheliomas.^90^ Finally, an ongoing phase 2 trial is examining oral sirolimus in combination with cyclophosphamide for the treatment of advanced sarcoma.^91^

**Table 1:** Phase 2 and 3 Studies of Mammalian Target of Rapamycin Inhibitors in Patients With Sarcoma

Agent (Phase)	Reference	Study Type	Malignancy	No. of Patients	Formulation	Comment
Sirolimus (2)	Bissler 2008^85^	Nonrandomized, open-label, 24-mo study (sirolimus only for first 12 mo)	Angiomyolipoma with TSC or sporadic lymphangioleiomyomatosis	25	Oral	Moderate regression of tumor size
Temsirolimus (2)	Okuno 2011^8^, ^6^	Multicenter, open-label study	Advanced metastatic STS	41	IV	Acceptable toxicity profile: Failed to demonstrate promising activity in patients with advanced STS
Everolimus (2)	Schoffski 2010^87^	Two-stage study with 2 strata: First-line failure and postsecond line	Treatment-experienced, imatinib-resistant GIST	28	Oral	Stratum 1 study stopped after first stage; PFS, 17%-37% at 4 mo
Ridaforolimus (2)	Chawla (in press)^88^	Nonrandomized, single-agent, open-label study	Treatment-experienced advanced STS and bone sarcoma	212	IV	Overall clinical benefit, 29%; median OS, 40 wk
Ridaforolimus (3)	Chawla 2011^89^	Randomized, double-blind, placebo- controlled study of maintenance therapy	Metastatic sarcoma	711	Oral	Median PFS, 17.7 wk with ridaforolimus vs 14.6 wk with placebo (HR, 0.72; *P* = .0001); Median OS, 88 wk with ridaforolimus vs 78.7 wk with placebo

Abbreviations: GIST, gastrointestinal stromal tumor; HR, hazard ratio; IV, intravenous; mTOR, mammalian target of rapamycin; OS, overall survival; PFS, progression-free survival; STS, soft tissue sarcoma; TSC, tuberous sclerosis complex.

#### Temsirolimus

A multicenter, phase 2 study evaluated weekly intravenous temsirolimus in chemotherapy-naive patients (N = 41) with advanced metastatic STS but failed to meet its clinical endpoints. Among 38 evaluable patients, 2 patients achieved a confirmed PR, including 1 patient with fibrosarcoma and another patient with leiomyosarcoma (Table 1).^86^ The median time to progression was estimated at 2 months (95% confidence interval, 1.8-3.5 months). Most patients experienced AEs, with 43% of patients experiencing grade 3/4 events at least possibly related to treatment. Although these results indicate that treatment with temsirolimus alone does not seem to be a promising therapy for patients with advanced STS, it is important to note that the study endpoint was a confirmed tumor response to treatment, defined as a CR or PR on 2 consecutive evaluations at least 4 weeks apart.^86^ The exclusion of SD in the assessment of treatment outcome resulted in a lower treatment response rate compared with other trials in sarcoma that evaluated other clinical endpoints, such as clinical benefit response, which incorporates SD. Another phase 2 trial examined intravenous temsirolimus in 52 pediatric patients with recurrent/refractory neuroblastoma, high-grade glioma, or rhabdomyosarcoma.^92^ Preliminary data from that trial indicated that 2 patients (1 neuroblastoma, 1 rhabdomyosarcoma) achieved a PR at 12 weeks and that 11 patients achieved SD that lasted for ≥12 weeks.^92^ Although the trial failed to meet its endpoint of tumor response (at least 2 patients in a subgroup needed to experience objective responses once 12 patients in that group had been enrolled), the responses observed and the clinical benefit attained by some patients suggest that further assessment may be warranted.

Several ongoing phase 2 trials are evaluating the benefit of intravenous temsirolimus in patients with various subtypes of sarcoma. Temsirolimus is being investigated as a single agent in patients with STS or GIST^93^ as well as patients with recurrent or persistent uterine cancer.^94^ Also, temsirolimus is being evaluated in combination studies with vinorelbine and cyclophosphamide in patients with recurrent or refractory rhabdomyosarcoma,^95^ and with selumetinib, a mitogen-activated protein kinase kinase (MEK) inhibitor, in patients with metastatic, recurrent, or locally advanced unresectable STS.^93^

#### Everolimus

The oral agent everolimus has been studied as a combination therapy in a phase 2 trial in patients with imatinib-resistant GIST. All patients received everolimus (2.5 mg daily) and imatinib (600 mg daily) (Table 1).^87^ Patients were enrolled in 2 strata: those who progressed after first-line treatment with oral imatinib and those who progressed after imatinib and other therapies (most patients received oral sunitinib as second-line treatment). Of the 28 patients in the study who failed prior treatment with imatinib, 23 were evaluable, and 4 of those patients (17.4%) were progression-free at 4 months. In addition, 47 patients enrolled in the trial had failed treatment with first-line imatinib and second-line sunitinib; among the 35 patients who were evaluable, 13 (37.1%) were progression-free at 4 months. Most patients reported AEs: Sixty-seven percent experienced grade 3 or 4 AEs, and 48% experienced SAEs. These results suggest that patients with GIST may benefit from combined treatment in case of first-line and second-line treatment failure. In another phase 2 study, everolimus was studied in patients with STS or bone sarcoma, but limited clinical efficacy was observed (CR/PR or SD rate, 20%). The most common AEs were skin toxicity, mucositis, and fatigue; serious AEs included pneumonitis and anemia.^96^

Everolimus is being investigated in 2 other phase 2 trials: 1) a multicenter, triple-arm trial investigating everolimus monotherapy in patients with progressive or metastatic STS or bone sarcoma and in patients with GIST who failed treatment with first-line oral imatinib or second-line oral sunitinib^97^; and 2) a single-arm, open-label monotherapy trial in patients with resectable STS of the extremities or the retroperitoneum.^98^ An ongoing phase 2/3 trial is further evaluating the benefit of combined treatment with everolimus and oral imatinib in patients with progressive GIST.^99^

#### Ridaforolimus

In a phase 2, open-label, nonrandomized trial, patients with advanced sarcomas (N = 212) received a 12.5-mg intravenous dose of ridaforolimus administered daily for 5 days every 2 weeks (Table 1).^89^ The overall rate of patients achieving a clinical benefit was 29%, including 4 patients who had a confirmed PR (2 osteosarcomas, 1 spindle cell sarcoma, and 1 malignant fibrous histiocytoma) and 3 patients who had an unconfirmed PR (1 osteosarcoma, 1 small round cell desmoplastic sarcoma, and 1 unclassifiable STS). The median overall survival (OS) was 40 weeks. The use of clinical benefit response to assess treatment outcome produced a higher treatment response rate compared with the temsirolimus trial described above, which used the confirmed objective response rate. Appropriate clinical trial endpoints to evaluate treatment outcomes in sarcoma have not been fully established and are a current topic of debate. All patients reported at least 1 treatment-emergent AE, and 21 SAEs were reported as at least possibly related to treatment in 20 patients. An ongoing phase 2 study is designed to assess the benefit of oral ridaforolimus in patients with metastatic bone or STS.^100^

### Phase 3 Studies

**Figure 2:**
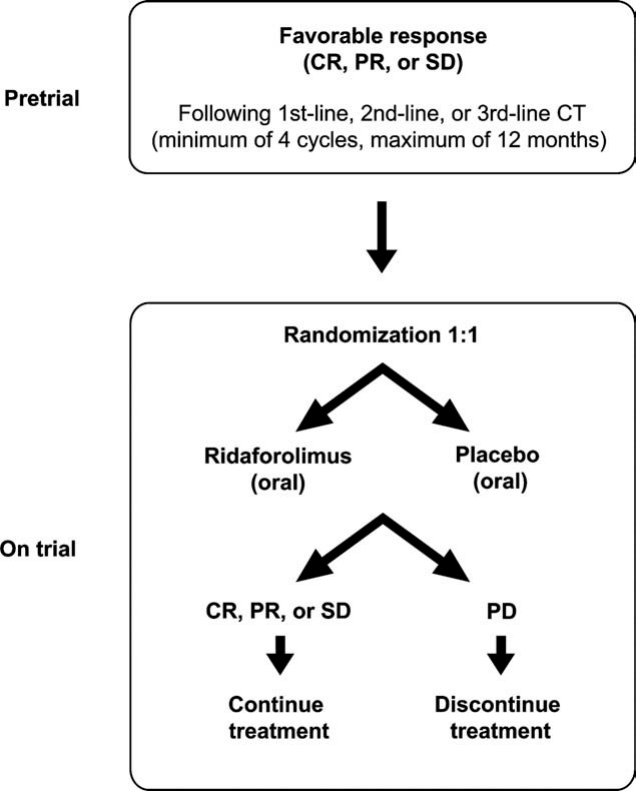
The Sarcoma Multicenter Clinical Evaluation of the Efficacy of Ridaforolimus (SUCCEED) study design is illustrated. CR, indicates complete response; PR, partial response; SD, stable disease; CT, chemotherapy; PD, progressive disease.

On the basis of results from the phase 1 oral study in metastatic solid tumors and the phase 2 intravenous study in sarcoma, an oral formulation of ridaforolimus at a dose of 40 mg once daily 5 times per week was selected for testing in a large phase 3 study in patients with sarcoma. The Sarcoma Multicenter Clinical Evaluation of the Efficacy of Ridaforolimus (SUCCEED) trial was designed to determine whether oral ridaforolimus can be used to maintain disease stability in the metastatic setting.^101^ The multicenter, multinational, double-blind, placebo-controlled, randomized, phase 3 trial was planned to evaluate 650 patients with metastatic sarcoma who have had favorable outcomes (eg, SD, PR, or CR) to first-line, second-line, or third-line chemotherapy. The primary outcome measure is PFS; secondary efficacy endpoints include OS, best target lesion response, improvement in symptoms, and safety and tolerability (Fig. .2).^101^ Top-line data recently presented from the SUCCEED trial demonstrate that treatment with oral ridaforolimus resulted in a 28% reduction (*P* = .0001) in the risk of progression compared with placebo (hazard ratio, 0.72) and a statistically significant 21% (3.1 week) improvement in median PFS (ridaforolimus vs placebo, 17.7 weeks vs 14.6 weeks).^89^ In a preliminary analysis based on 313 events, the median OS with ridaforolimus was 88.0 weeks versus 78.7 weeks in the placebo group. The incidence of stomatitis (52%) and other AEs was higher with ridaforolimus than with placebo; these findings were consistent with safety data reported for other mTOR inhibitors. Although additional data on secondary endpoints are pending, including updated OS data, these initial results for using ridaforolimus in the treatment of STS seem promising.

### Safety and Tolerability in Phase 2 and 3 Trials

Table 2 summarizes safety data from phase 2 and 3 studies of the mTOR inhibitors in patients with advanced metastatic sarcomas (temsirolimus, ridaforolimus), imatinib-resistant GIST (everolimus in combination with imatinib), or angiomyolipomas (sirolimus).^85^-^89^ The most common AEs reported for at least 2 mTOR inhibitors include mouth ulcers (characterized as apthous ulcers, mucositis, or stomatitis), diarrhea, fatigue, anemia, and nausea. Mucositis/stomatitis is the most common dose-limiting toxicity of these agents; the inflammation of the oral mucosa associated with mTOR inhibitors is distinct from conventional mucositis and appears to have a different underlying mechanism.^102^ In addition to oral-related side effects, other mTOR class-specific AEs of clinical relevance include metabolic/laboratory abnormalities—such as hyperlipidemia and hypokalemia—skin disorders, and pneumonitis.^85^, ^89^ Severe or grade 3/4 AEs reported across multiple studies with mTOR inhibitors include stomatitis, vomiting, fatigue, anemia, and hypokalemia. Overall, mTOR inhibitors generally have demonstrated acceptable safety and tolerability profiles in clinical trials.

**Table 2:** Safety Summary From Phase 2 and 3 Trials of Mammalian Target of Rapamycin Inhibitors

Agent	Reference	Most Common Adverse Events: All Grades	Grade 3/4 or Serious Adverse Events
Sirolimus	Bissler 2008^85^	Apthous ulcers, diarrhea, upper respiratory infections	Diarrhea, pyelonephritis, stomatitis, respiratory infections^a^
Temsirolimus	Okuno 2011^86^	Stomatitis, fatigue, anemia	Stomatitis, fatigue, nausea, vomiting, dyspnea^b^
Everolimus (in combination with imatinib)	Schoffski 2010^87^	Diarrhea, nausea, fatigue, anemia	Hypokalemia, anemia, lymphopenia, fatigue, vomiting^b^
Ridaforolimus	Chawla (in press), Chawla 2011^88^, ^89^	Mucositis/stomatitis, fatigue, hypertriglyceridemia, anemia, rash, nausea	Anemia, thrombocytopenia, hypophosphatemia, hyponatremia, hypokalemia^b^

a Serious adverse events.

b Grade 3/4 adverse events.

### Future Prospects

Recent in vitro evidence has indicated that pretreating sarcoma cells with an IGF-1R antibody blocks rapamycin-induced feedback activation of Akt, thereby leading to enhanced cytotoxicity.^11^ On the basis of this evidence, a phase 1 study using a combination of oral everolimus and intravenous figitumumab, an antibody to IGF-1R, in patients with advanced sarcomas demonstrated that 83% of patients who were evaluable for a response achieved SD.^103^ Similarly, another phase 1 study currently is evaluating oral ridaforolimus in combination with intravenous dalotuzumab, an investigational monoclonal antibody that also blocks IGF-1R, in patients with advanced solid tumors.^104^ Intravenous temsirolimus also is being studied in a phase 2 trial in combination with intravenous cixutumumab, another monoclonal antibody to IGF-1R, for the treatment of sarcomas, including recurrent STS or bone sarcoma and Ewing sarcoma.^105^, ^106^ Several orally available, novel ATP-competitive mTOR inhibitors currently are in phase 1 and 2 trials, including NVP-BEZ235 (Novartis), a dual PI3K/mTOR modulator that blocks the dysfunctional activation of the PI3K pathway and induces G1 arrest,^107^ and the PI3K inhibitors XL765 (Exelixis) and XL147.^5^ Although the benefit observed with current mTOR inhibitors seems to be primarily in maintaining SD, the second generation of mTOR inhibitors currently being developed may have the potential to produce higher objective tumor response rates.

In conclusion, currently, a very limited number of treatment options exist for sarcomas. Mammalian target of rapamycin, a serine/threonine kinase, has a pivotal role in controlling cell growth, metabolism, cell proliferation, angiogenesis, and cell survival. Dysregulation of mTOR-associated signaling pathways has been observed in several malignancies, including sarcomas. Consequently, the mTOR pathway is considered an important target for anticancer drug development. Clinical studies of mTOR inhibitors have demonstrated encouraging results across a broad range of tumor types and present promising treatment options, particularly for patients with advanced sarcoma, whose tumors are challenging to treat. Class-specific AEs observed with mTOR inhibitors include mild-to-moderate mouth ulcers (described as either stomatitis, mucositis, or aphthouse-like), skin rash/erythema, and some metabolic abnormalities. The mTOR inhibitors currently under clinical investigation for use in the treatment of patients with advanced or metastatic sarcoma include sirolimus, temsirolimus, everolimus, and ridaforolimus, all of which have demonstrated a favorable toxicity profile and antitumor activity in a variety of sarcoma subtypes.

## FUNDING SOURCES

Editorial support was funded by Merck.

## CONFLICT OF INTEREST DISCLOSURES

The author has participated in advisory boards for Novartis Pharmaceuticals and has received honoraria from Novartis Pharmaceuticals.

## References

[1] National Comprehensive Cancer Network (2011). NCCN Clinical Practice Guidelines in Oncology™: Soft Tissue Sarcoma, Version 1.2011.

[2] Surveillance Epidemiology and End Results Program (2010). SEER Stat Fact Sheet: Soft Tissue Including Heart.

[3] Surveillance Epidemiology and End Results Program (2010). SEER Stat Fact Sheet: Bone and Joint.

[4] Mahalingam D, Mita A, Sankhala K (2009). Targeting sarcomas: novel biological agents and future perspectives. Curr Drug Targets.

[5] Fasolo A, Sessa C (2008). mTOR inhibitors in the treatment of cancer. Expert Opin Investig Drugs.

[6] Goberdhan DC, Boyd CA (2009). mTOR: dissecting regulation and mechanism of action to understand human disease. Biochem Soc Trans.

[7] Jiang BH, Liu LZ (2008). Role of mTOR in anticancer drug resistance: perspectives for improved drug treatment. Drug Resist Updat.

[8] Figlin RA, Brown E, Armstrong AJ (2008). NCCN Task Force Report: mTOR inhibition in solid tumors. J Natl Compr Canc Netw.

[9] Faivre S, Kroemer G, Raymond E (2006). Current development of mTOR inhibitors as anticancer agents. Nat Rev Drug Discov.

[10] Dancey JE (2006). Therapeutic targets: mTOR and related pathways. Cancer Biol Ther.

[11] Mahalingam D, Sankhala K, Mita A, Giles FJ, Mita MM (2009). Targeting the mTOR pathway using deforolimus in cancer therapy. Future Oncol.

[12] Wan X, Helman LJ (2007). The biology behind mTOR inhibition in sarcoma. Oncologist.

[13] Menon S, Manning BD (2008). Common corruption of the mTOR signaling network in human tumors. Oncogene.

[14] Memmott RM, Dennis PA (2009). Akt-dependent and -independent mechanisms of mTOR regulation in cancer. Cell Signal.

[15] Averous J, Proud CG (2006). When translation meets transformation: the mTOR story. Oncogene.

[16] Hay N, Sonenberg N (2004). Upstream and downstream of mTOR. Genes Dev.

[17] Bjornsti MA, Houghton PJ (2004). The TOR pathway: a target for cancer therapy. Nat Rev Cancer.

[18] Ma XM, Blenis J (2009). Molecular mechanisms of mTOR-mediated translational control. Nat Rev Mol Cell Biol.

[19] Semenza GL (2003). Targeting HIF-1 for cancer therapy. Nat Rev Cancer.

[20] Rafii S, Lyden D, Benezra R, Hattori K, Heissig B (2002). Vascular and haematopoietic stem cells: novel targets for anti-angiogenesis therapy?. Nat Rev Cancer.

[21] Hsieh AC, Moasser MM (2007). Targeting HER proteins in cancer therapy and the role of the non-target HER3. Br J Cancer.

[22] Yuan R, Kay A, Berg WJ, Lebwohl D (2009). Targeting tumorigenesis: development and use of mTOR inhibitors in cancer therapy [serial online]. J Hematol Oncol.

[23] Chitnis MM, Yuen JS, Protheroe AS, Pollak M, Macaulay VM (2008). The type 1 insulin-like growth factor receptor pathway. Clin Cancer Res.

[24] Samani AA, Yakar S, LeRoith D, Brodt P (2007). The role of the IGF system in cancer growth and metastasis: overview and recent insights. Endocr Rev.

[25] Xie Y, Skytting B, Nilsson G, Brodin B, Larsson O (1999). Expression of insulin-like growth factor-1 receptor in synovial sarcoma: association with an aggressive phenotype. Cancer Res.

[26] Girnita L, Girnita A, Wang M, Meis-Kindblom JM, Kindblom LG, Larsson O (2000). A link between basic fibroblast growth factor (bFGF) and EWS/FLI-1 in Ewing's sarcoma cells. Oncogene.

[27] Hughes DP, Thomas DG, Giordano TJ, Baker LH, McDonagh KT (2004). Cell surface expression of epidermal growth factor receptor and Her-2 with nuclear expression of Her-4 in primary osteosarcoma. Cancer Res.

[28] Ganti R, Skapek SX, Zhang J (2006). Expression and genomic status of EGFR and ErbB-2 in alveolar and embryonal rhabdomyosarcoma. Mod Pathol.

[29] Sampson JR (2009). Therapeutic targeting of mTOR in tuberous sclerosis. Biochem Soc Trans.

[30] Umeoka S, Koyama T, Miki Y, Akai M, Tsutsui K, Togashi K (2008). Pictorial review of tuberous sclerosis in various organs [serial online]. Radiographics.

[31] Boyd KP, Korf BR, Theos A (2009). Neurofibromatosis type 1. J Am Acad Dermatol.

[32] Katz D, Lazar A, Lev D (2009). Malignant peripheral nerve sheath tumour (MPNST): the clinical implications of cellular signalling pathways. Expert Rev Mol Med.

[33] Kapoor A, Figlin RA (2009). Targeted inhibition of mammalian target of rapamycin for the treatment of advanced renal cell carcinoma. Cancer.

[34] Ritacco FV, Graziani EI, Summers MY (2005). Production of novel rapamycin analogs by precursor-directed biosynthesis. Appl Environ Microbiol.

[35] Dudkin L, Dilling MB, Cheshire PJ (2001). Biochemical correlates of mTOR inhibition by the rapamycin ester CCI-779 and tumor growth inhibition. Clin Cancer Res.

[36] DeGraffenried LA, Fulcher L, Friedrichs WE, Grunwald V, Ray RB, Hidalgo M (2004). Reduced PTEN expression in breast cancer cells confers susceptibility to inhibitors of the PI3 kinase/Akt pathway. Ann Oncol.

[37] Kirchner GI, Meier-Wiedenbach I, Manns MP (2004). Clinical pharmacokinetics of everolimus. Clin Pharmacokinet.

[38] Pfizer (2008). TORISEL (temsirolimus) prescribing information.

[39] Novartis Pharmaceuticals Corporation (2010). Afinitor (everolimus) prescribing information.

[40] Mita M, Sankhala K, Abdel-Karim I, Mita A, Giles F (2008). Deforolimus (AP23573) a novel mTOR inhibitor in clinical development. Expert Opin Investig Drugs.

[41] Busca R, Bertolotto C, Ortonne JP, Ballotti R (1996). Inhibition of the phosphatidylinositol 3-kinase/p70(S6)-kinase pathway induces B16 melanoma cell differentiation. J Biol Chem.

[42] Grewe M, Gansauge F, Schmid RM, Adler G, Seufferlein T (1999). Regulation of cell growth and cyclin D1 expression by the constitutively active FRAP-p70s6K pathway in human pancreatic cancer cells. Cancer Res.

[43] Houghton PJ, Morton CL, Kolb EA (2008). Initial testing (stage 1) of the mTOR inhibitor rapamycin by the pediatric preclinical testing program. Pediatr Blood Cancer.

[44] Houghton PJ, Morton CL, Gorlick R (2010). Stage 2 combination testing of rapamycin with cytotoxic agents by the Pediatric Preclinical Testing Program. Mol Cancer Ther.

[45] Wan X, Shen N, Mendoza A, Khanna C, Helman LJ (2006). CCI-779 inhibits rhabdomyosarcoma xenograft growth by an antiangiogenic mechanism linked to the targeting of mTOR/Hif-1alpha/VEGF signaling. Neoplasia.

[46] Mitsiades N, McMullan C, Poulaki V (2004). The mTOR inhibitor RAD001 (everolimus) is active against multiple myeloma cells in vitro and in vivo [abstract]. Blood (ASH Annual Meeting Abstracts).

[47] Johnson BE, Jackman D, Janne PA (2007). Rationale for a phase I trial of erlotinib and the mammalian target of rapamycin inhibitor everolimus (RAD001) for patients with relapsed nonsmall cell lung cancer. Clin Cancer Res.

[48] Villanueva A, Chiang DY, Newell P (2008). Pivotal role of mTOR signaling in hepatocellular carcinoma. Gastroenterology.

[49] Mabuchi S, Altomare DA, Connolly DC (2007). RAD001 (everolimus) delays tumor onset and progression in a transgenic mouse model of ovarian cancer. Cancer Res.

[50] O'Reilly T, Vaxelaire J, Muller M (2002). In vivo activity of RAD001, an orally active rapamycin derivative, in experimental tumor models [abstract]. Proc Am Assoc Cancer Res.

[51] Boulay A, Zumstein-Mecker S, Stephan C (2004). Antitumor efficacy of intermittent treatment schedules with the rapamycin derivative RAD001 correlates with prolonged inactivation of ribosomal protein S6 kinase 1 in peripheral blood mononuclear cells. Cancer Res.

[52] Majewski M, Korecka M, Joergensen J (2003). Immunosuppressive TOR kinase inhibitor everolimus (RAD) suppresses growth of cells derived from posttransplant lymphoproliferative disorder at allograft-protecting doses. Transplantation.

[53] Boulay A, Rudloff J, Ye J (2005). Dual inhibition of mTOR and estrogen receptor signaling in vitro induces cell death in models of breast cancer. Clin Cancer Res.

[54] Rossi F, Ehlers I, Agosti V (2006). Oncogenic Kit signaling and therapeutic intervention in a mouse model of gastrointestinal stromal tumor. Proc Natl Acad Sci U S A.

[55] Hernando E, Charytonowicz E, Dudas ME (2007). The AKT-mTOR pathway plays a critical role in the development of leiomyosarcomas. Nat Med.

[56] Clackson T, Metcalf CA, Rivera VM (2003). Broad anti-tumor activity of ap23573, an mTOR inhibitor in clinical development [abstract]. Proc Am Soc Clin Oncol.

[57] Squillace R, Miller D, Clapham D (2008). Anti-tumor activity of the mTOR inhibitor deforolimus (AP23573; MK-8669) in sarcoma and endometrial cancer models and exploration of molecular correlates of sensitivity [abstract 1482].

[58] Squillace R, Miller D, Clackson T, Rivera V (2008). Anti-proliferative activity of the mTOR inhibitor deforolimus (AP23573; MK-8669) in combination with cytotoxic and targeted agents in sarcoma and endometrial cancer models [abstract 4006].

[59] Jimeno A, Rudek MA, Kulesza P (2008). Pharmacodynamic-guided modified continuous reassessment method-based, dose-finding study of rapamycin in adult patients with solid tumors. J Clin Oncol.

[60] AIDS Malignancy Clinical Trials Consortium (2011). Sirolimus in treating patients with HIV-related Kaposi's sarcoma. NCT00450320.

[61] Cohen EE, Sharma M, Janisch LA (2010). A phase I study of sirolimus (rapamycin) and bevacizumab in patients with advanced malignancies [abstract]. J Clin Oncol.

[62] Cirstea D, Hideshima T, Rodig S (2010). Dual inhibition of akt/mammalian target of rapamycin pathway by nanoparticle albumin-bound-rapamycin and perifosine induces antitumor activity in multiple myeloma. Mol Cancer Ther.

[63] Abraxis BioScience, LLC (2011). ABI-009 trial in patients with advanced non-hematologic malignancies. NCT00635284.

[64] Hidalgo M, Buckner JC, Erlichman C (2006). A phase I and pharmacokinetic study of temsirolimus (CCI-779) administered intravenously daily for 5 days every 2 weeks to patients with advanced cancer. Clin Cancer Res.

[65] Raymond E, Alexandre J, Faivre S (2004). Safety and pharmacokinetics of escalated doses of weekly intravenous infusion of CCI-779, a novel mTOR inhibitor, in patients with cancer. J Clin Oncol.

[66] Buckner JC, Forouzesh B, Erlichman C (2010). Phase I, pharmacokinetic study of temsirolimus administered orally to patients with advanced cancer. Invest New Drugs.

[67] Cancer Therapy and Research Center, Texas, Sorafenib and temsirolimus in treating patients with unresectable or metastatic solid tumors (2011). http://clinicaltrials.gov/ct2/show/NCT00255658?term=NCT00255658&rank=1.

[68] University of North Carolina Lineberger Comprehensive Center (2011). Temsirolimus and valproic acid in treating young patients with relapsed neuroblastoma, bone sarcoma, or soft tissue sarcoma. NCT01204450.

[69] University of Southern California/Norris Comprehensive Cancer Center (2011). Temsirolimus and vinorelbine ditartrate in treating patients with unresectable or metastatic solid tumors. NCT01155258.

[70] Sidney Kimmel Comprehensive Cancer Center (2011). Safety and efficacy study of torisel and liposomal doxorubicin for patients with recurrent sarcoma. NCT00949325.

[71] New Mexico Cancer Care Alliance (2011). Phase I/II study of irinotecan and temsirolimus in patients with refractory sarcomas. NCT00996346.

[72] Tabernero J, Rojo F, Calvo E (2008). Dose- and schedule-dependent inhibition of the mammalian target of rapamycin pathway with everolimus: a phase I tumor pharmacodynamic study in patients with advanced solid tumors. J Clin Oncol.

[73] O'Donnell A, Faivre S, Burris HA (2008). Phase I pharmacokinetic and pharmacodynamic study of the oral mammalian target of rapamycin inhibitor everolimus in patients with advanced solid tumors. J Clin Oncol.

[74] Seeliger H, Guba M, Kleespies A, Jauch KW, Bruns CJ (2007). Role of mTOR in solid tumor systems: a therapeutical target against primary tumor growth, metastases, and angiogenesis. Cancer Metastasis Rev.

[75] Fouladi M, Laningham F, Wu J (2007). Phase I study of everolimus in pediatric patients with refractory solid tumors. J Clin Oncol.

[76] Hospital Santa Marcelina (2011). Phase II study of everolimus in children and adolescents with refractory or relapsed rhabdomyosarcoma and other soft tissue sarcomas. NCT01216839.

[77] St. Jude Children's Research Hospital (2011). Everolimus for treating pediatric patients with recurrent or refractory tumors. NCT00187174.

[78] Mita MM, Mita AC, Chu QS (2008). Phase I trial of the novel mammalian target of rapamycin inhibitor deforolimus (AP23573; MK-8669) administered intravenously daily for 5 days every 2 weeks to patients with advanced malignancies. J Clin Oncol.

[79] Hartford CM, Desai AA, Janisch L (2009). A phase I trial to determine the safety, tolerability, and maximum tolerated dose of deforolimus in patients with advanced malignancies. Clin Cancer Res.

[80] Mita MM, Britten CD, Poplin E (2008). Deforolimus trial 106—a phase I trial evaluating 7 regimens of oral deforolimus (AP23573, MK-8669) [abstract]. J Clin Oncol.

[81] Gore L, Trippett TM, Katzenstein HM (2010). A multicenter, first-in-pediatrics phase I study of ridaforolimus (AP23573, MK-8669) in patients (pts) with refractory solid tumors [abstract]. J Clin Oncol.

[82] Sessa C, Tosi D, Vigano L (2009). Phase Ib study of weekly mammalian target of rapamycin inhibitor ridaforolimus (AP23573; MK-8669) with weekly paclitaxel. Ann Oncol.

[83] Merck (2010). Trial of deforolimus in combination with bevacizumab for patients with advanced cancers. NCT00781846.

[84] The University of Texas Health Science Center at San Antonio (2011). Study of oral ridaforolimus in combination with standard chemotherapy for soft tissue sarcoma. NCT01296659.

[85] Bissler JJ, McCormack FX, Young LR (2008). Sirolimus for angiomyolipoma in tuberous sclerosis complex or lymphangioleiomyomatosis. N Engl J Med.

[86] Okuno S, Bailey H, Mahoney MR (2011). A phase 2 study of temsirolimus (CCI-779) in patients with soft tissue sarcomas: a study of the Mayo Phase 2 Consortium (P2C) [published online ahead of print February 1, 2011]. Cancer.

[87] Schoffski P, Reichardt P, Blay JY (2010). A phase I-II study of everolimus (RAD001) in combination with imatinib in patients with imatinib-resistant gastrointestinal stromal tumors. Ann Oncol.

[88] Chawla SP, Staddon AP, Baker LH A phase 2 study of the mTOR inhibitor ridaforolimus (AP23573; MK 8669) in patients with advanced bone and soft tissue sarcomas. J Clin Oncol.

[89] Chawla SP, Blay J, Ray-Coquard IL (2011). Results of the phase III, placebo-controlled trial (SUCCEED) evaluating the mTOR inhibitor ridaforolimus (R) as maintenance therapy in advanced sarcoma patients (pts) following clinical benefit from prior standard cytotoxic chemotherapy (CT) [abstract]. J Clin Oncol.

[90] Children's Hospital Medical Center, Cincinnati (2011). Safety and efficacy study of sirolimus in complicated vascular anomalies. NCT00975819.

[91] University of Michigan Cancer Center (2011). A phase II study of oral cyclophosphamide and sirolimus (OCR) in advanced sarcoma. NCT00743509.

[92] Geoerger B, Kieran MW, Grupp S (2010). Phase II study of temsirolimus in children with high-grade glioma, neuroblastoma, and rhabdomyosarcoma [abstract]. J Clin Oncol.

[93] Beckman Research Institute (2011). MEK inhibitor AZD6244 with or without temsirolimus in treating patients with metastatic, recurrent, or locally advanced soft tissue sarcoma that cannot be removed by surgery. NCT01206140.

[94] Albert Einstein College of Medicine of Yeshiva University (2011). Temsirolimus in treating patients with recurrent or persistent cancer of the uterus. NCT01061606.

[95] Children's Oncology Group (2011). Vinorelbine ditartrate and cyclophosphamide in combination with bevacizumab or temsirolimus in treating patients with recurrent or refractory rhabdomyosarcoma. NCT01222715.

[96] Richter S, Pink D, Hohenberger P (2010). Multicenter, triple-arm, single-stage, phase II trial to determine the efficacy and safety of everolimus (RAD001) in patients with refractory bone or soft tissue sarcomas including GIST [abstract]. J Clin Oncol.

[97] Novartis Pharmaceuticals (2011). Treatment of patients with RAD001 who have progressive sarcoma. NCT00767819.

[98] Dana-Farber Cancer Institute (2011). RAD001 in combination with CP-751,871 in patients with advanced sarcomas and other malignant neoplasms. NCT00927966.

[99] Novartis Pharmaceuticals (2011). Treatment of patients with everolimus and imatinib mesylate who have progressive gastrointestinal stromal tumors (GIST) and are resistant to imatinib mesylate. NCT00510354.

[100] Merck (2011). Phase II study of MK8669 with metastatic bone or soft-tissue sarcoma patients (8669–030). NCT01010672.

[101] Merck (2010). Ridaforolimus in treatment of sarcoma—SUCCEED (Sarcoma Multi-Center Clinical Evaluation of the Efficacy of Ridaforolimus). http://clinicaltrials.gov/ct2/show/NCT00538239?term=ridaforolimus&rank=8.

[102] Sonis S, Treister N, Chawla S, Demetri G, Haluska F (2010). Preliminary characterization of oral lesions associated with inhibitors of mammalian target of rapamycin in cancer patients. Cancer.

[103] Quek R, Wang Q, Morgan JA (2011). Combination mTOR and IGF-1R inhibition: phase I trial of everolimus and figitumumab in patients with advanced sarcomas and other solid tumors. Clin Cancer Res.

[104] Merck (2010). A combination study with ridaforolimus (MK8669) and dalotuzumab (MK0646) in patients with advanced cancer. NCT00730379.

[105] Memorial Sloan-Kettering Cancer Center (2011). Temsirolimus and cixutumumab in treating patients with locally advanced, metastatic, or recurrent soft tissue sarcoma or bone sarcoma. NCT01016015.

[106] Naing A, LoRusso P, Gupta S (2010). Dual inhibition of IGFR and mTOR pathways [abstract]. J Clin Oncol.

[107] Manara MC, Nicoletti G, Zambelli D (2010). NVP-BEZ235 as a new therapeutic option for sarcomas. Clin Cancer Res.

